# Selective etching of silicon nitride over silicon oxide using ClF_3_/H_2_ remote plasma

**DOI:** 10.1038/s41598-022-09252-3

**Published:** 2022-04-05

**Authors:** Won Oh Lee, Ki Hyun Kim, Doo San Kim, You Jin Ji, Ji Eun Kang, Hyun Woo Tak, Jin Woo Park, Han Dock Song, Ki Seok Kim, Byeong Ok Cho, Young Lae Kim, Geun Young Yeom

**Affiliations:** 1 School of Advanced Materials Science and Engineering, Sungkyunkwan University, 2066 Seobu-ro, Jangan-gu, Suwon-si, Gyeonggi-do 16419 Republic of Korea; 2 Research and Development Group, Wonik Materials Co. Ltd., Cheongju, 28125 Republic of Korea; 3 Research Laboratory of Electronics, Massachusetts Institute of Technology, Cambridge, MA USA; 4 SKKU Advanced Institute of Nano Technology (SAINT), Sungkyunkwan University, 2066 Seobu-ro, Jangan-gu, Suwon-si, Gyeonggi-do 16419 Republic of Korea

**Keywords:** Chemical engineering, Composites

## Abstract

Precise and selective removal of silicon nitride (SiN_x_) over silicon oxide (SiO_y_) in a oxide/nitride stack is crucial for a current three dimensional NOT-AND type flash memory fabrication process. In this study, fast and selective isotropic etching of SiN_x_ over SiO_y_ has been investigated using a ClF_3_/H_2_ remote plasma in an inductively coupled plasma system. The SiN_x_ etch rate over 80 nm/min with the etch selectivity (SiN_x_ over SiO_y_) of ~ 130 was observed under a ClF_3_ remote plasma at a room temperature. Furthermore, the addition of H_2_ to the ClF_3_ resulted in an increase of etching selectivity over 200 while lowering the etch rate of both oxide and nitride due to the reduction of F radicals in the plasma. The time dependent-etch characteristics of ClF_3_, ClF_3_ & H_2_ remote plasma showed little loading effect during the etching of silicon nitride on oxide/nitride stack wafer with similar etch rate with that of blank nitride wafer.

## Introduction

As the semiconductor device size is decreased to sub-nanoscale and the device integration is changed from two dimensional to three dimensional structure, more precise and selective etch technology is required for the semiconductor device fabrication^1^. In the various semiconductor devices, silicon nitride has been widely used as a barrier layer for dopant diffusion, a gate sidewall spacer layer, a buffer layer, etc. due to high insulating characteristics, high thermal and mechanical stability, etc. and selective etching of silicon nitride over silicon and/or silicon oxide is important for various microelectronic applications^2^.

These days, in the three dimensional NOT-AND type flash memory fabrication, the number of silicon nitride/silicon oxide (SiN_x_/SiO_y_) stack is increasing and the thickness of repeating SiN_x_/SiO_y_ layer is decreasing continuously for higher memory density in the vertical direction. Therefore, the etching of SiN_x_ layers uniformly and ultra-high selectively to SiO_y_ layers in the SiN_x_/SiO_y_ stack is becoming more challenging process. Until now, the selective etching of SiN_x_ in SiN_x_/SiO_y_ stacks is achieved by wet etching using a hot phosphoric acid (H_3_PO_4_)^3–6^. In case of the wet etching, however, the penetration of an etch solution into holes is getting more challenging as the thickness of the SiN_x_/SiO_y_ layer is decreased and the remaining SiO_y_ layers can be collapsed due to the surface tension. Moreover, several additives for increasing the etch selectivity of SiN_x_/SiO_y_ are found to cause oxide regrowth problems after etching unless its process condition is not carefully controlled^5^. To solve these problems, a dry process for isotropic and selective etching of SiN_x_ needs to be developed as an alternative technology for three dimensional NOT-AND type flash memory fabrication.

Various studies have been reported for selective etching of SiN_x_ over SiO_y_ using dry etch processes. For example, an ultra-high selective etching of SiN_x_ over SiO_y_ was reported using CF_4_-based (CF_4_/O_2_/N_2_, CF_4_/CH_4_/Ar) gases with a microwave chemical downstream etcher and an inductively coupled plasma (ICP) etcher^7–9^. In addition, NF_3_-based (NF_3_/O_2_/NH_3_, NF_3_/O_2_/N_2_) gases were also used to ultra-high selective etching of silicon nitride over silicon oxide with downstream etchers based on ICP or capacitively coupled plasma (CCP)^9–13^. However, the etch selectivity of nitride over oxide still needs to be increased further for the application of current semiconductor process due to the thin thickness of oxide. Moreover, the use of fluorocarbon (CF_x_) etch gases has contamination issues by carbon or deposition of CF_x_ (CH_x_) polymers on the surface of the film, and which is a detrimental factor for a device fabrication. Even though these limits for engineering aspects are excluded, the high global warming potentials (GWPs) of CF_4_- and NF_3_-based etch gases [GWP values; CF_4_ (7,390), NF_3_ (17,200)] arouse the needs for the alternative etch gases for environmental aspects in the near future^14^.

ClF_3_ with the GWP of ~ 0 has been used primarily as an in-situ cleaning gas for chemical vapor deposition (CVD) chambers in replacement of perfluorocarbon compounds (PFC), which have high GWP values or as an etch gas for silicon etching by heating, neutral cluster beam etching, reactive ion beam etching, etc.^15–19^. In addition, the ClF_3_ have been investigated for etching of SiGe in an ICP system^20^, SiC etching with ultra-high etch rate over 10 µm/min^21^, selective etching of transition metals and metal nitrides such as tantalum (tantalum nitride) over metal oxide (Ta_2_O_5_) with low pressure gaseous etching method^22^. In this study, ClF_3_ remote plasma was applied for a fast and ultra-high selective etching of silicon nitride (SiN_x_) over silicon oxide (SiO_y_) applicable for current and next-generation semiconductor device fabrication including three dimensional NOT-AND type flash memory. The etching of SiN_x_ using ClF_3_ showed high etch rate over 80 nm/min and the etch selectivity of SiN_x_ over SiO_y_ of ~ 130. The etch selectivity of SiN_x_ was further increased with H_2_ addition in the ClF_3_ plasma. The effect of Cl, F, and H radicals on the selective etching of SiN_x_ was investigated using plasma and surface analysis tools, and its etch mechanism was suggested.

## Experimental section

### Etching of silicon nitride

**Figure 1:**
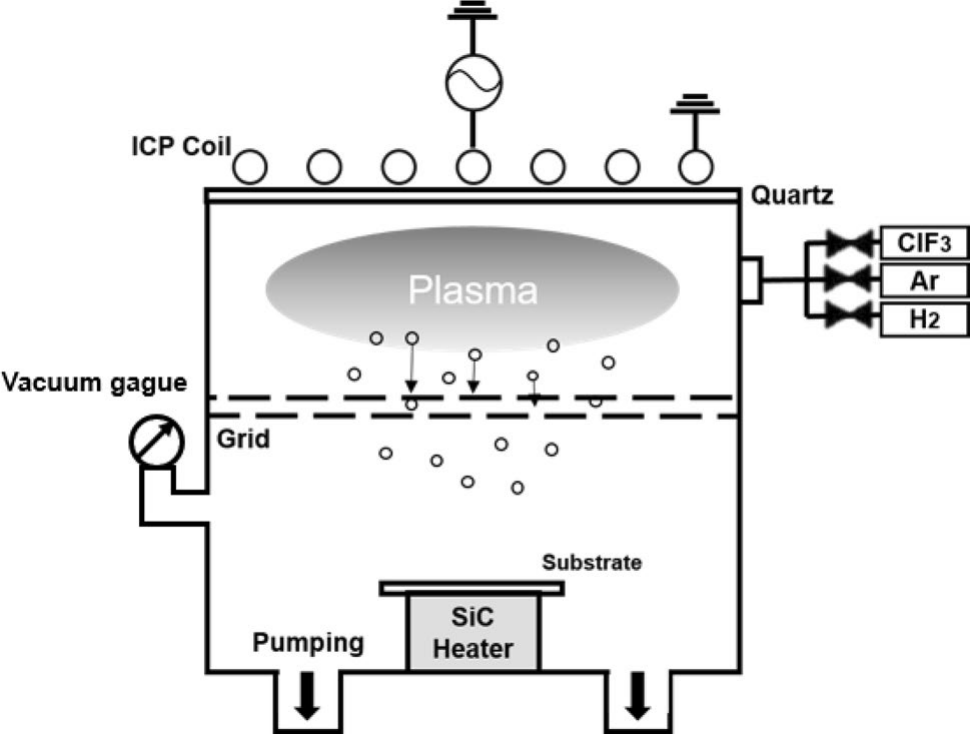
Schematic drawing of a remote-type inductively coupled plasma (ICP) etcher. At the center of the chamber, double grids having multiple holes are installed to prevent an ion bombardment and to deliver radicals only to the substrate. During the process, the substrate temperature was controlled (RT ~ 500 °C) by a silicon carbide (SiC) heater located below the substrate.

Figure 1 is a schematic drawing of a remote type inductively coupled plasma (ICP) etching system used in this study. The inside of process chamber was coated with an aluminum oxide layer by anodizing. The base pressure of the process chamber measured with a convection gauge was maintained at 3 × 10^–3^ Torr and the operating pressure monitored by capacitance manometer (Baratron gauge) was maintained at 200 mTorr. 13.56 MHz RF power was applied to the planar type ICP coil at upper side of a chamber. For the isotropic etching of SiN_x_, double grids with multiple holes with 1.5 mm radius were arranged at the center of ICP reactor to prevent an ion bombardment effect and deliver radicals on the substrate. The substrate temperature was measured at the sample stage below the sample, which was monitored by a thermocouple and adjusted from 25 to 500 °C by a silicon carbide (SiC) heater connected to an external power supply. The chlorine trifluoride (ClF_3_, > 99.9%, 200 sccm), H_2_ (> 99.999%), and Argon (> 99.999% Ar, 200 sccm) were flown through a circular shape gas distributor to the process chamber.

### Sample preparation

Blank 1.5 µm thick SiN_x_ thin films, blank 300 nm thick SiO_y_ thin films, and multilayer stacks composed of repeating SiO_y_ (27 nm) and SiN_x_ (27 nm) thin films were deposited by a plasma enhanced chemical vapor deposition (PECVD) process (supplied by WONIK IPS Inc.).

### Characterization

The etch rate of SiN_x_ and SiO_y_ were measured by a step profilometer (Tencor, Alpha-step 500) and with Scanning Emission Microscopy (SEM, Hitachi S-4700) after patterning with photoresist (PR, AZ 5214E) as an etch mask. Also, the etch profiles of the multilayer thin films composed of SiN_x_/SiO_y_ stacks were observed by the SEM. The surface roughness of films after the etching was measured by atomic force microscope (AFM, XE-100, Park System) with a non-contact measurement mode. The characteristics of ClF_3_/H_2_ plasma were analyzed with Optical Emission Spectrometry (OES, Avaspec-3648). Byproduct gases during etching process were monitored with Fourier-Transform Infrared Spectroscopy (FT-IR, MIDAC 12,000). The binding state and atomic composition of SiN_x_ and SiO_y_ (thin films of initial thickness of 500, 300 nm, respectively) before and after the etching were analyzed by X-ray Photoelectron Spectroscopy (XPS, MXP10, ThermoFisher Scientific) with a monochromated Al Kα source (1,486.6 eV) with spot size of 400 µm. The expected energy resolution of XPS is below 0.5 eV FWHM. The Avantage 5.0 software was used for curve fittings and the areas of each peak were calculated with shirley background. The incident angle of X-ray to the sample was 50° and a hemispherical sector energy analyzer was positioned perpendicular to the sample stage.

## Results and discussion

**Figure 2:**
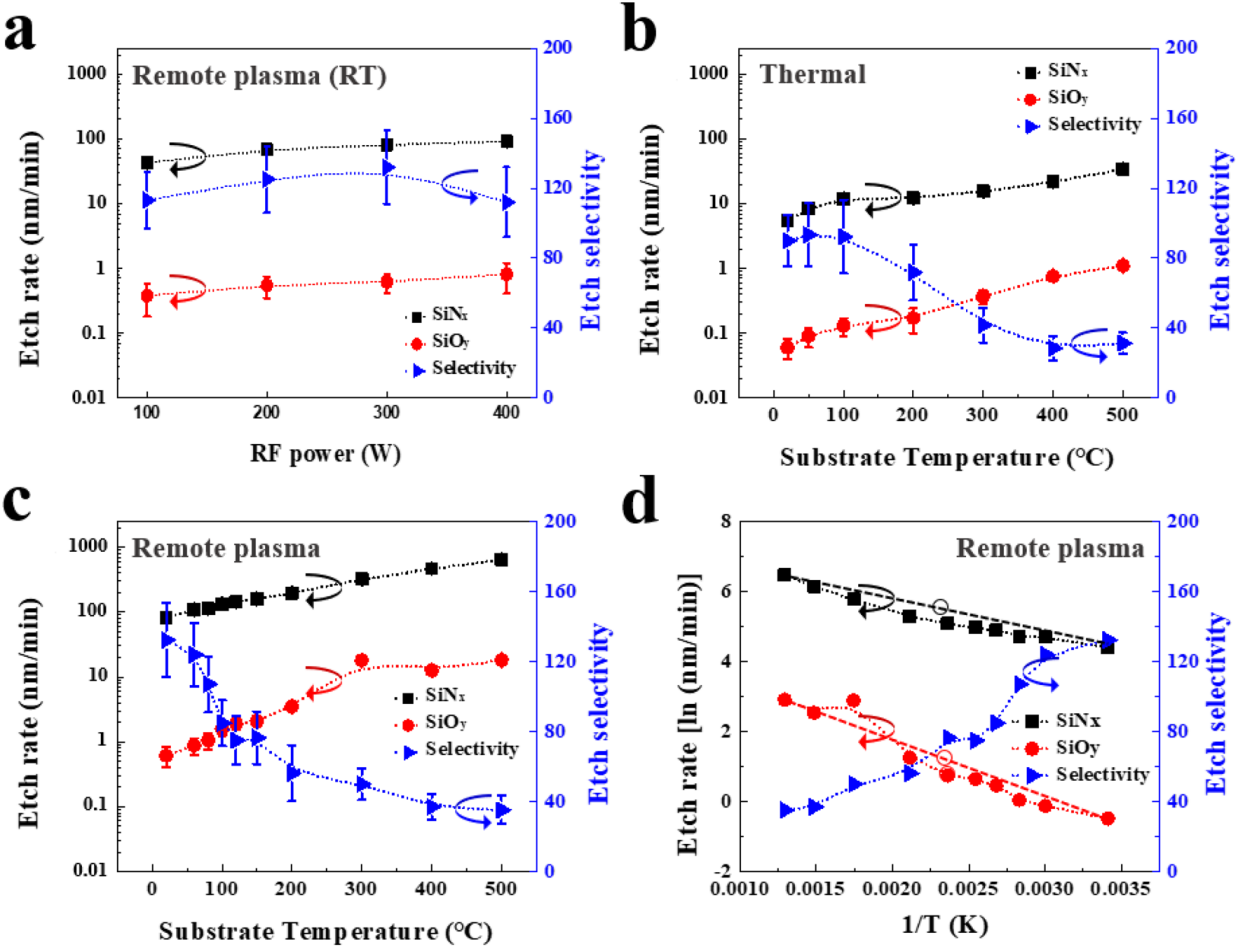
Etch characteristics of SiN_x_ and SiO_y_ (**a**) as a function of rf power for ClF_3_ remote plasma at RT, (**b**) as a function of substrate temperature for chemical etching with ClF_3_ gas flow only, and (**c**) as a function of substrate temperature for ClF_3_ remote plasma at 300 W of rf power. 200 sccm Ar (200 sccm) was added to ClF_3_ for plasma stability. (**d**) logarithm etch rates versus 1/T for ClF_3_ remote plasma etching of SiN_x_ and SiO_y_ in (**c**) for the extraction of the activation energies.

Figure 2 shows etch characteristics of SiN_x_ and SiO_y_ with ClF_3_ gas only and ClF_3_ remote plasmas. For ClF_3_ remote plasmas, 200 sccm of Ar was added to 200 sccm of ClF_3_ for the plasma stability. As shown in Fig. 2a, the etch rates of SiN_x_ and SiO_y_ were increased gradually with increasing rf power due to the enhanced dissociation of ClF_3_ reaching the maximum etch rates of SiN_x_ and SiO_y_ at ~ 90 and ~ 0.8 nm/min, respectively. Note that, the etch selectivity of SiN_x_ over SiO_y_ didn’t vary significantly (~ 120) over rf powers of 100 ~ 400 W. As shown in Fig. 2b, the SiN_x_ and SiO_y_ could be also etched just by flowing ClF_3_ gas only without dissociating ClF_3_ by rf plasmas and the increase of substrate temperature increased the etch rates of both films. However, the overall SiN_x_ etch rates by ClF_3_ gas flow only were much lower compared to etching with ClF_3_ remote plasmas, and which demonstrates that ClF_3_ remote plasma etching is much more effective method for SiN_x_ etching compared with that by thermal etching without plasma. Meanwhile, even though etch rates of both materials were increased with increasing the substrate temperature, the etch selectivity of SiN_x_ over SiO_y_ was decreased. Same trend was observed for remote plasma etching. As shown in Fig. 2c, the increase of substrate temperature to ~ 500 °C at a fixed rf power of 300 W showed a gradual decreases of etch selectivity below 40 while showing increased SiN_x_ etch rates over 600 nm/min. The effect of process temperature on the etching of SiN_x_ and SiO_y_ can be understood by plotting the etch rates of SiN_x_ and SiO_y_ logarithmically as a function of inverse temperature (1/T) for ClF_3_ remote plasma etching as shown in Fig. 2d. For the chemically activated etching, the etch rates can be described as a following Arrhenius equation.

$$\ln k = - \frac{{E_{a} }}{R}\left( {\frac{1}{{T_{2} }} - \frac{1}{{T_{1} }}} \right)$$

where *k* is a rate constant, *R* is the gas constant (1.987 cal K^-1^ mol^-1^), T is the process temperature (K), and *E*_*a*_ is the activation energy. The calculated activation energies (*E*_*a*_) of SiN_x_ and SiO_y_ were 1.93 and 3.18 kcal/mole, respectively. The higher activation energy of SiO_y_ means that the etch rate of SiO_y_ rises faster than that of SiN_x_ with the increase of temperature, and which leads to the decreases in etch selectivity of SiN_x_ over SiO_y_ even though the etch rates of both materials are increased exponentially with increasing substrate temperature. The root mean square (RMS) surface roughness of SiN_x_ and SiO_y_ after the etching with each process condition (remote plasma- and thermally-etching) showed no significant differences in the RMS surface roughness among the samples for different etch methods (Figure S1, supplementary information).

**Figure 3:**
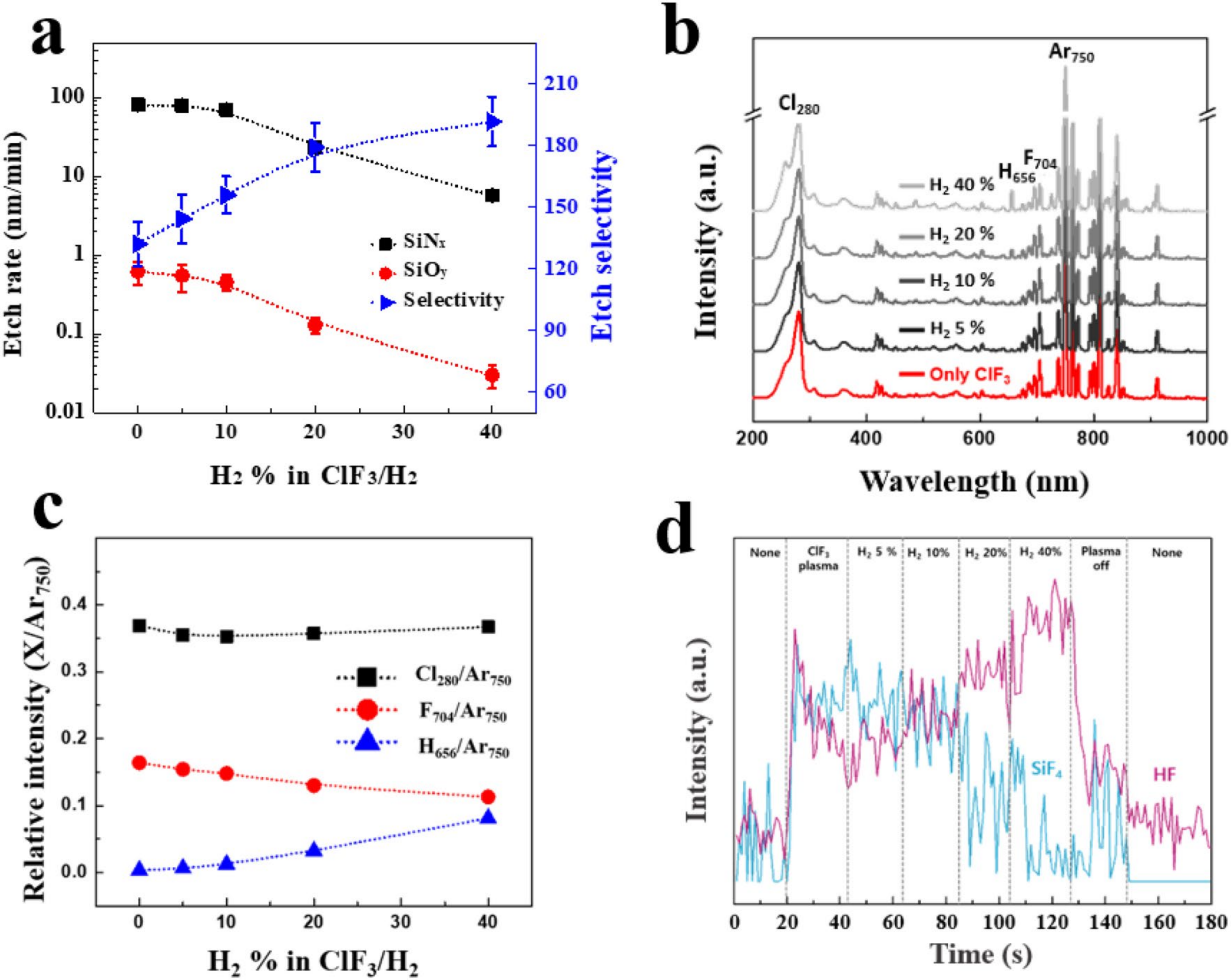
(**a**) Etch characteristics of SiN_x_ and SiO_y_ with ClF_3_/H_2_ plasma as a function of H_2_ percentage in ClF_3_/H_2_. (**b**) OES data of ClF_3_/H_2_/Ar plasma with different H_2_ percentage in ClF_3_/H_2_. (**c**) optical emission intensities of Cl, F, and H normalized by the intensity of Ar (750 nm) in (**b**) plotted as a function of H_2_ percentage. (**d**) FTIR data of ClF_3_/H_2_ plasma during SiN_x_ etching. For ClF_3_/H_2_ remote plasmas, 200 sccm of Ar was added for the plasma stability.

To improve the etch selectivity of SiN_x_ over SiO_y_, H_2_ was added to ClF_3_ in addition to Ar (Ar was also added to ClF_3_/H_2_ for plasma stability) and, the effect of H_2_ addition to ClF_3_ on the etch characteristics of SiN_x_ and SiO_y_ was investigated as a function of H_2_ percentage in ClF_3_/H_2_ (ClF_3_/H_2_/Ar plasma) and the results are shown in Fig. 3a. To increase the H_2_ percentage in ClF_3_/H_2_, H_2_ flow rate was increased while keeping the substrate temperature at 25 °C, operating pressure at 200 mTorr, the ClF_3_ flow rate at 200 sccm, Ar flow rate at 200 sccm, and the rf power at 300 W. The etch rates of both SiN_x_ and SiO_y_ were decreased with the increase of H_2_ percentage, however, the etch selectivity of SiN_x_ over SiO_y_ was increased with the increase of H_2_ percentage in ClF_3_/H_2_. To study the mechanism of selectivity SiN_x_ etching over SiO_y_, the dissociated species in the plasmas was investigated by OES at the center of chamber and the byproducts during the process was monitored using FTIR at the pumping site. Figure 3b,c shows optical emission spectra and the relative emission peak intensities of Cl, F, and H normalized by the intensity of Ar as a function of H_2_ percentage in ClF_3_/H_2_, respectively. In Fig. 3b, the optical emission peak intensities related to Cl, H, F, and Ar could be measured at 280, 656, 704, and 750 nm, respectively. In Fig. 3c, the optical emission intensities of Cl, F, and H were normalized by the optical emission intensity of Ar (750 nm) to minimize the effect of electron density on the estimation of radical density from the emission intensity. As shown in Fig. 3c, the increase of H_2_ percentage did not change the intensity of Cl, however, it decreased F intensity while increasing H intensity. Figure 3 shows the FTIR data of the byproduct gases such as SiF_4_ and HF measured at the pumping site for different H_2_ percentage in ClF_3_/H_2_. As the flow rate of H_2_ is increased, the concentration of SiF_4_ was decreased, and which means that the etching of SiN_x_ was suppressed while increasing HF concentration due to the reaction of hydrogen (H) with fluorine (F) radical in the plasma. Usually, the addition of hydrogen to fluorine based plasma leads to scavenging of F radicals by forming gaseous HF molecules ^23,24^ which have negligible effects on the etching of SiN_x_ (and SiO_y_) unlike their aqueous (ionized) state^25,26^.

**Figure 4:**
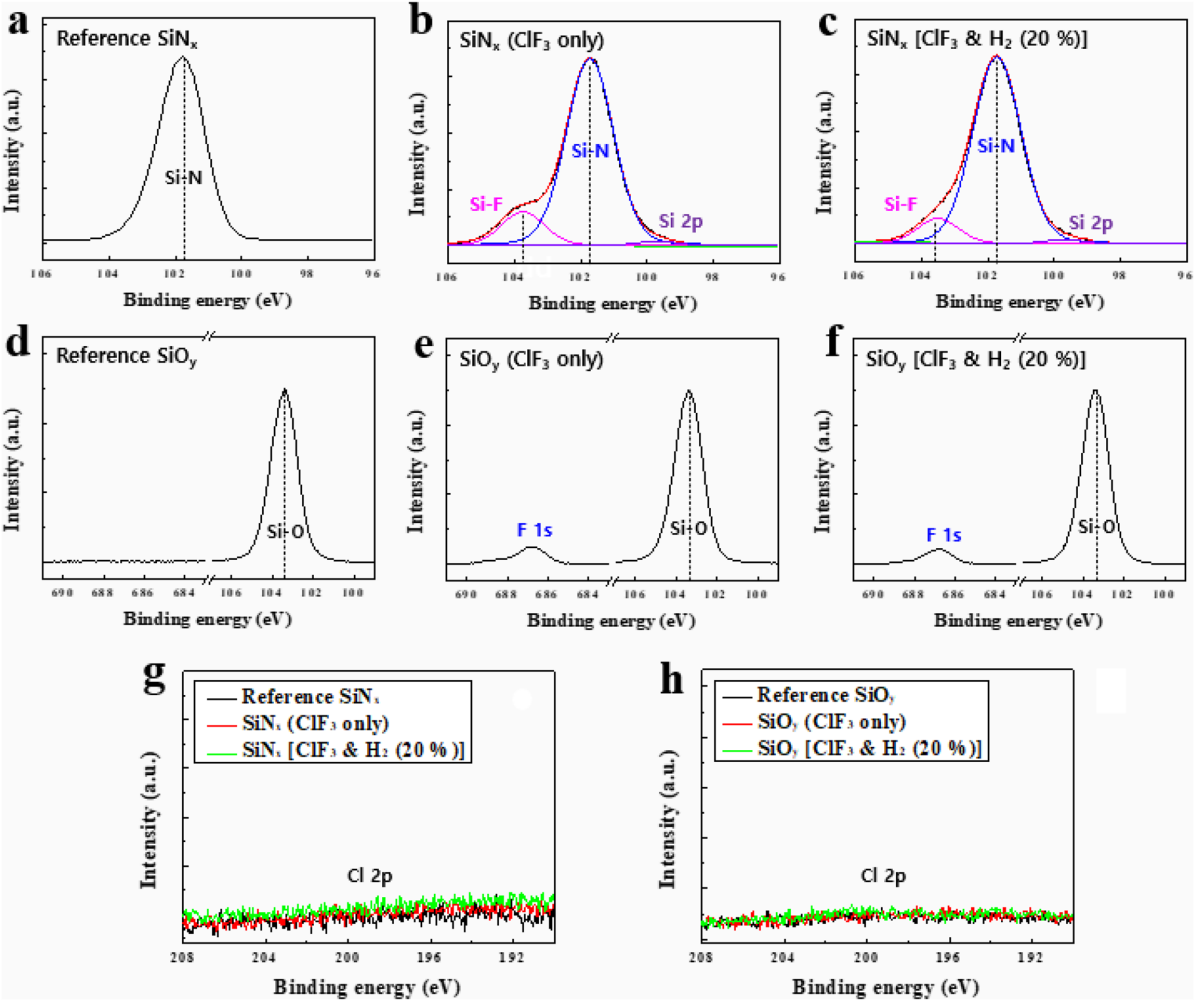
XPS narrow scan (Si 2p) data of SiN_x_ (**a**-**c**), SiO_y_ (**d**-**f**), and Cl 2p (**g**, **h**) after etching with a remote ClF_3_/H_2_ plasma.

The Si binding states and surface composition of SiN_x_ and SiO_y_ after the ClF_3_/H_2_ plasma etching were analyzed using X-ray Photoelectron Spectroscopy (XPS) and the results are shown in Fig. 4 and Table 1. The SiN_x_ and SiO_y_ were etched at the substrate temperature of 25 °C, operating pressure at 200 mTorr, the ClF_3_/H_2_/Ar flow rates at 200/(0 and 40)/200 sccm, and the rf power at 300 W. As shown in Fig. 4a,d, the reference SiN_x_ and SiO_y_ showed only Si–N at 101.7 eV, Si–O at 103.4 eV, respectively. After the etching with ClF_3_ plasma, however, significant Si–F bonding (103.6 eV) was formed on the SiN_x_ surface, presumably due to the bonding of Si with F (Fig. 4b). The Si–F bonding ratio decreases with addition of H_2_ (20%) because of the reduction of F in the plasma (Fig. 4c and Table 1). However, no chlorine or Si-Cl bonding (~ 103.3 eV) was observed on the surface of SiN_x_ even though there were enough Cl radicals in the ClF_3/_H_2_ plasma as confirmed through OES data in Fig. 3b, presumably, due to the immediate reaction of Si-Cl with F radicals. Meanwhile, as shown in Fig. 4e,f), there was no significant change in F concentration on the SiO_y_ surface during etching with ClF_3_ and ClF_3_/H_2_ plasma. Also, no noticeable Si–F bonding formation on the SiO_y_ surface during the etching with ClF_3_ and ClF_3_/H_2_ plasma was observed from the deconvolution of Si narrow scan data (Si 2p) indicating that most of F is adsorbed on the SiO_y_ surface after the etching. Furthermore, the amount of F on the SiO_y_ surface is much lower than that of SiN_x_ because Si–O bonding is less reactive with F radical compared with SiN_x_. As shown in Fig. 4g,h, no chlorine was observed on the surface of both SiN_x_ and SiO_y_ even though the chlorine was observed in OES (Fig. 3b). The parameters used for curve fitting of SiN_x_ is described in Table 1 and the normalized chi-square value for curve fitting was below 0.01. The compositional information of each element can be found in Table S1, supplementary information.

**Table 1:** Parameters related with the curve fitting of silicon nitride (SiN_x_) thin films after exposure to the ClF_3_ only and ClF_3_ & H_2_ (20%) plasma.

Sample	Binding state	B.E. (eV)	FWHM (eV)	% Area	Gaussian %
SiN_x_ (ClF_3_ only)	Si 2p	99.7	1.3	1.6	88.8
	Si–N	101.7	1.7	84.5	87.5
	Si–F	103.6	1.55 (± 0.05)	13.9	85.7
SiN_x_ [ClF_3_ & H_2_ (20%)]	Si 2p	99.7	1.3	1.6	88.8
	Si–N	101.7	1.7	88	87.5
	Si–F	103.6	1.55 (± 0.05)	10.4	85.7

**Figure 5:**
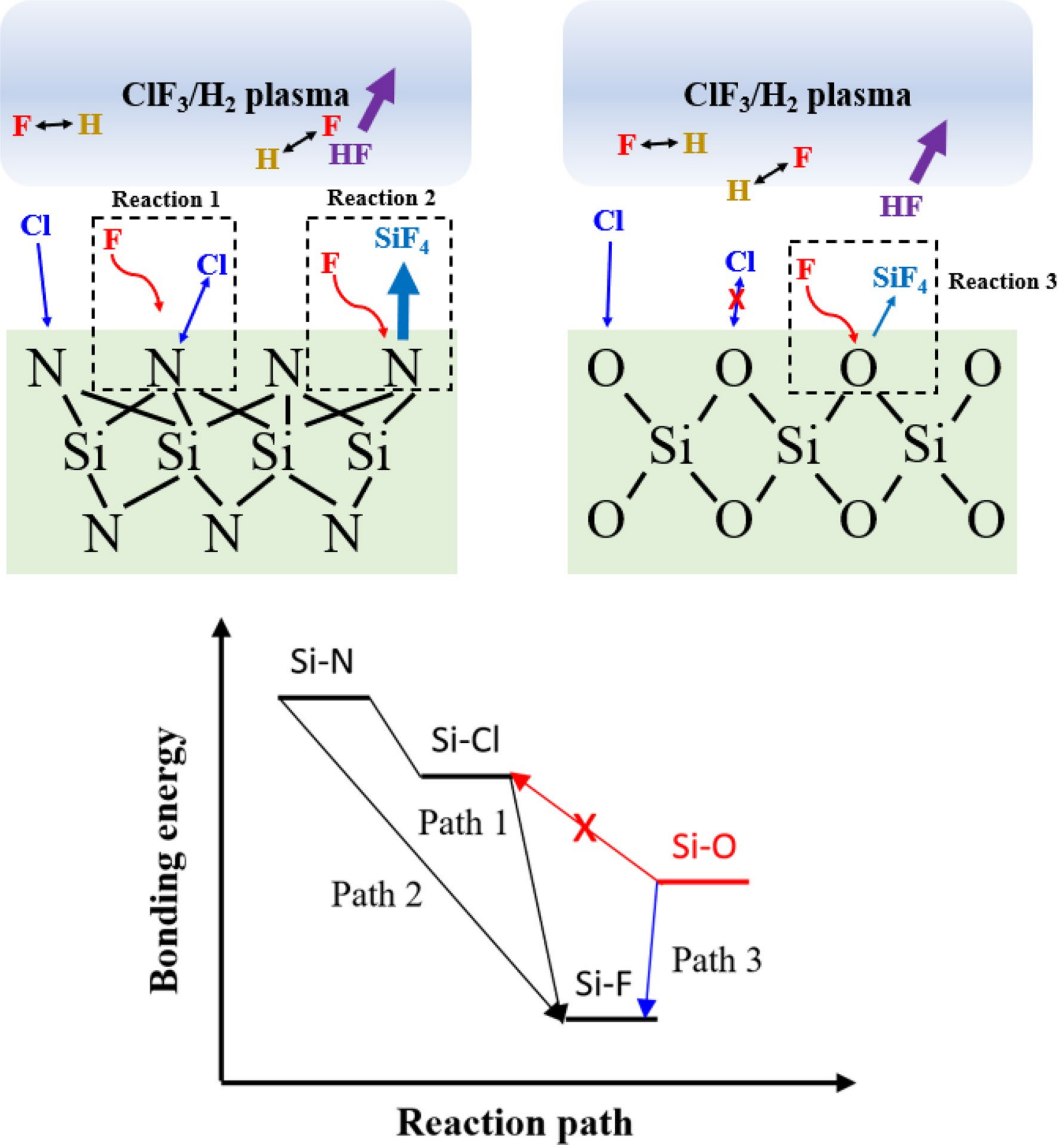
Schematic of chemical reaction of ClF_3_/H_2_ remote plasma on etching of SiN_x_ and SiO_y_. Possible reaction paths are illustrated.

The etching of SiN_x_ and SiO_y_ can be explained through the bonding energies of silicon (Si) compounds. Figure 5 shows the etch mechanism of SiN_x_ and SiO_y_ under Cl, F radicals. As the bonding energy of Si–F (565 kJ/mol) is higher than those of Si–N (355 kJ/mol) and Si–O (452 kJ/mol)^22^, the SiN_x_ and SiO_y_ can be etched spontaneously under sufficient F radicals in the plasma although the etching is much active for SiN_x_ than SiO_y_. However, the bonding energy of Si-Cl (381 kJ/mol) is slightly higher than that of Si–N but lower than that of Si–O, and which means the Cl radical can react only with SiN_x_ and forms Si-Cl bonding. Once the Si–N changes to Si-Cl, Si-Cl can be more easily converted to Si–F by F radicals in the plasma (due to the quick conversion of Si-Cl to Si–F as shown in Fig. 5, no chlorine could be observed on the surfaces of SiN_x_ and SiO_y_ during the etching with ClF_3_/H_2_), then Si–F on SiN_x_ is removed as a volatile SiF_4_ compound. Meanwhile, the addition of H_2_ in the ClF_3_ plasma reduces the density of F radicals by forming HF in the plasma causing the decreases of Si–F formation on the surfaces of SiN_x_ and SiO_y_, and which results in the decrease of etch rates of SiN_x_ and SiO_y_. However, because the concentration of chlorine in the plasma is not significantly affected by the addition of H_2_ as confirmed through OES data in Fig. 3c), the etching of SiN_x_ is decreased more slowly compared to that of SiO_y_ with increasing H_2_ percentage through the conversion of Si-Cl on the surface of SiN_x_ to Si–F, and which appears to increases the etch selectivity of SiN_x_ over SiO_y_.

**Figure 6:**
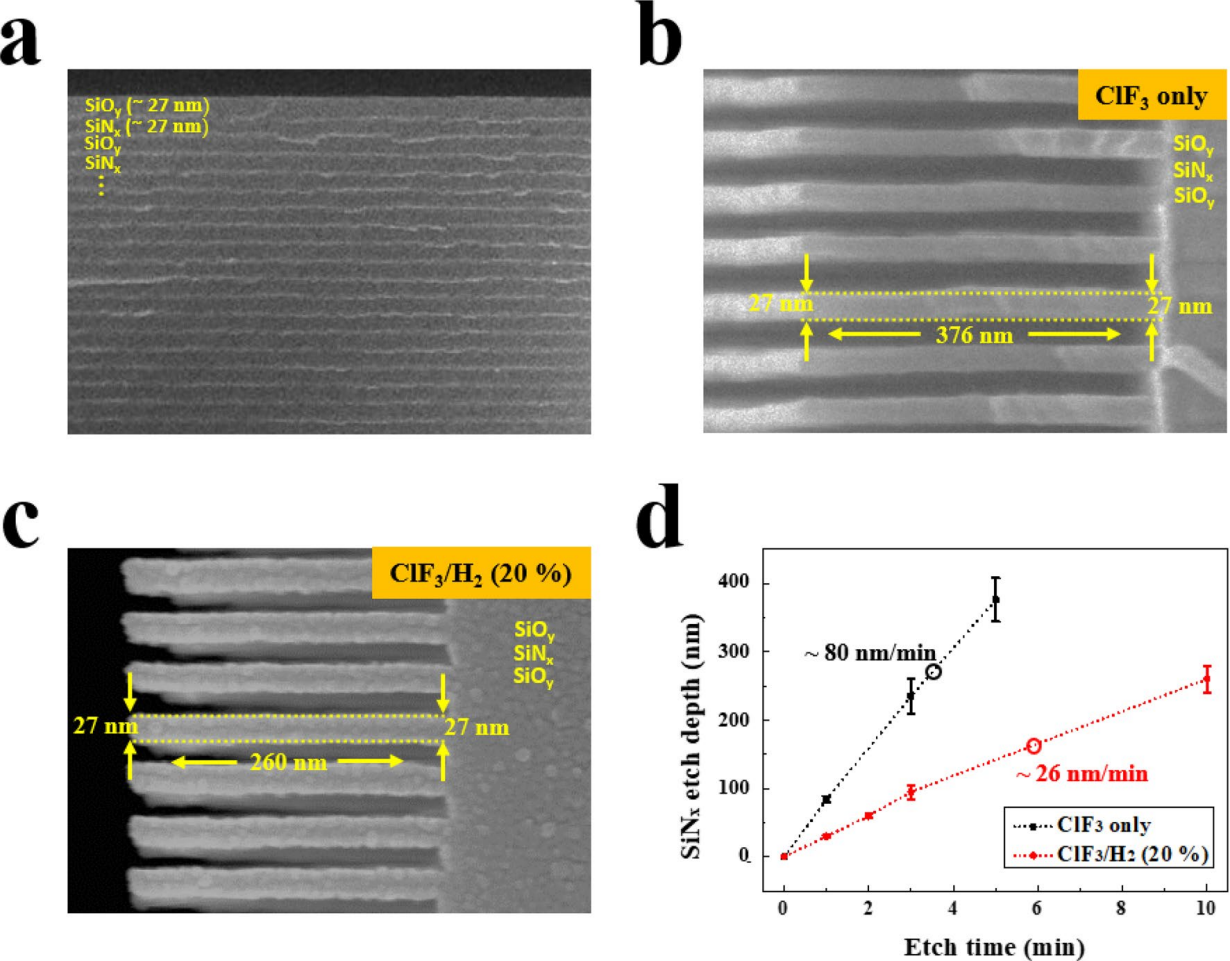
Etch characteristics of ClF_3_ only and ClF_3_/H_2_ (20%) plasma in stacked SiN_x_/SiO_y_. (**a**) SEM images of reference stacked SiN_x_/SiO_y_. Etch profile of stacked SiN_x_/SiO_y_ after the etching with (**b**) ClF_3_ plasma and (**c**) ClF_3_/H_2_ (20%) for 5 min and 10 min, respectively. (**d**) Etch depth of SiN_x_ in the stacked SiN_x_/SiO_y_ with etch time for ClF_3_ and ClF_3_/H_2_ (20%) plasmas.

Using the etch conditions of ClF_3_ and ClF_3_/H_2_ (20%), stacked layers of SiN_x_/SiO_y_ were etched and the results are shown in Fig. 6. Figure 6a is the reference stack of SiN_x_/SiO_y_ before the etching. Figure 6b,c are the stacked layer of SiN_x_/SiO_y_ after the etching using ClF_3_ and ClF_3_/H_2_ (20%) plasmas for 5 min and 10 min, respectively. As shown in Fig. 6b,c, highly selective etching of SiN_x_ over SiO_y_ could be observed for both ClF_3_ and ClF_3_/H_2_ (20%) by showing no noticeable differences in SiO_y_ thickness along the etch depth. Therefore, it appears that the etch selectivity for the real SiN_x_/SiO_y_ could be higher than that measured with blank wafers. The etch depth with increasing the etch time was also measured and the results are shown in d) for both ClF_3_ and ClF_3_/H_2_ (20%). The measured etch rates of SiN_x_ with ClF_3_ and ClF_3_/H_2_ remote plasma were 80 and 26 nm/min, respectively, which have similar values with blank samples at the same plasma conditions (Fig. 2a, 3a) because of isotropic etch characteristics of reactive radicals. Furthermore, the etch depth with etch time was linear for both conditions, therefore, no aspect ratio dependent etching was observed. (The process time-dependent etch profiles of SiN_x_/SiO_y_ stacks are shown in figure S2 and S3, supplementary information).

## Conclusion

The isotropic and selective etching of SiN_x_ over SiO_y_ was studied using ClF_3_/H_2_ remote plasma with an ICP source. The SiN_x_ etching using plasma assisted thermal processes showed the highest etch rate as well as the smoothest surface morphology compared with that etched only with thermal etching or plasma etching. The temperature dependent etch characteristics of SiN_x_ and SiO_y_ demonstrated a higher activation energy of SiO_y_ compared that of SiN_x_ in the ClF_3_ remote plasma. Furthermore, the addition of H_2_ (20%) to the ClF_3_ plasma improved the etch selectivity of SiN_x_ over SiO_y_ from 130 to 200 even though the etch rate of SiN_x_ was decreased from ~ 83 to ~ 23 nm/min. We believe the fast and ultra-high selective SiN_x_ etching technology can be applied not only to next generation three dimensional NOT-AND type flash memory fabrication process but also to various semiconductor processes where precise etching of SiN_x_ is required.

## Supplementary Information

Supplementary Information.
