# Supplementary material for: Selective etching of silicon nitride over silicon oxide using ClF3/H2 remote plasma
Source: Sci Rep. 2022 Apr 5;12:5703. doi: 10.1038/s41598-022-09252-3 (PMC8983696; doi:10.1038/s41598-022-09252-3)
Supplement: Supplementary file 1 — Supplementary Information. [file 41598_2022_9252_MOESM1_ESM.docx]

Selective etching of silicon nitride over silicon oxide using ClF_3_/H_2_ remote plasma


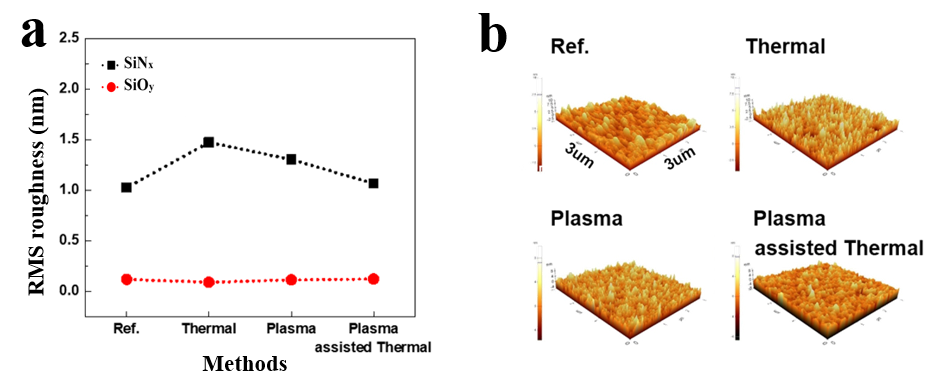


**Figure S1.** a) RMS surface roughness values and b) images of SiN_x_ and SiO_y_ after etching with ClF_3_ plasma [same plasma conditions with figure 3a) in the manuscript].

Figure S1 a) and b) show the RMS surface roughness values and the images of surface roughness of SiN_x_ and SiO_y_ after the etching with thermally with flowing ClF_3_, ClF_3_ plasma at room temperature, and ClF_3_ plasma with substrate temperature of 100 ℃. respectively. SiN_x_ and SiO_y_ were etched ~ 400 and ~ 20 nm, respectively. As shown in the figures, the surface roughness of etched SiN_x_ was the highest after the chemical etching with substrate temperature of 100 ℃ (1.5 nm) and it was followed by SiN_x_ after the remote plasma etching without substrate heating (1.3 nm). The lowest surface roughness which is similar to that of the reference SiN_x_ (~ 1 nm) was observed after the plasma etching at substrate heating with 100 ℃ (1.15 nm). Even though there were some differences in the surface roughness after the different etch methods, the differences are considered not significant. In the case of SiO_y_, possibly due to small etch rates for all of the cases, no significant change in the surface roughness was also observed after the etching with the different methods.


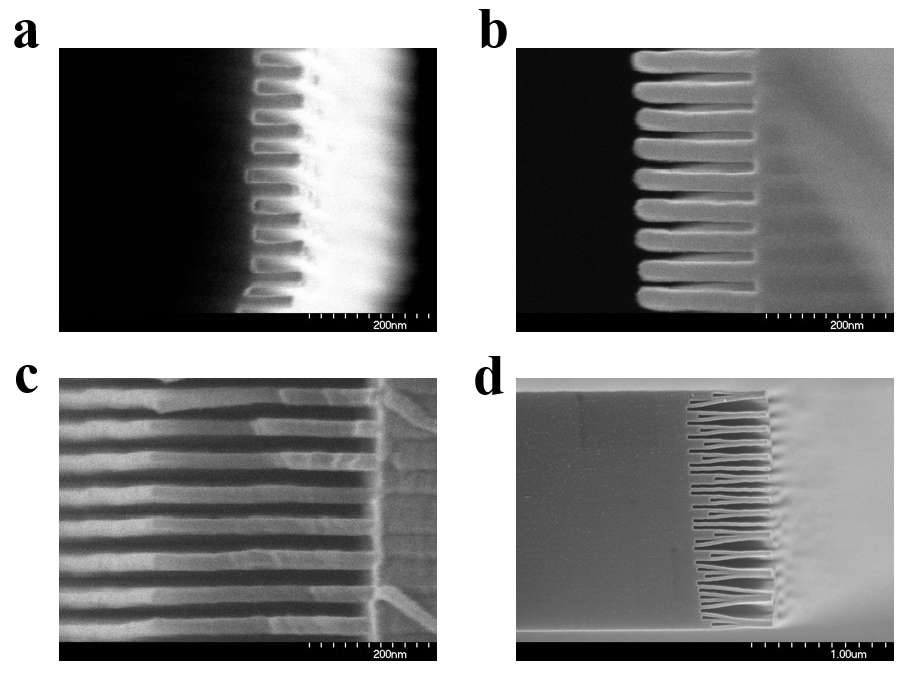


**Figure S2.** Cross-sectional SEM images of SiN_x_/SiO_y_ stacks during the etching with ClF_3_ plasma for a) 1, b) 3, c) 5, and d) 8 min, respectively.

During the process, the structure of SiN_x_/SiO_y_ stack started to collapse over 4000 Å-width SiN_x_ etch depth as shown in figure S2 d) but no noticeable changes in the remaining SiO_y_ thickness were observed up to 8 min etching.


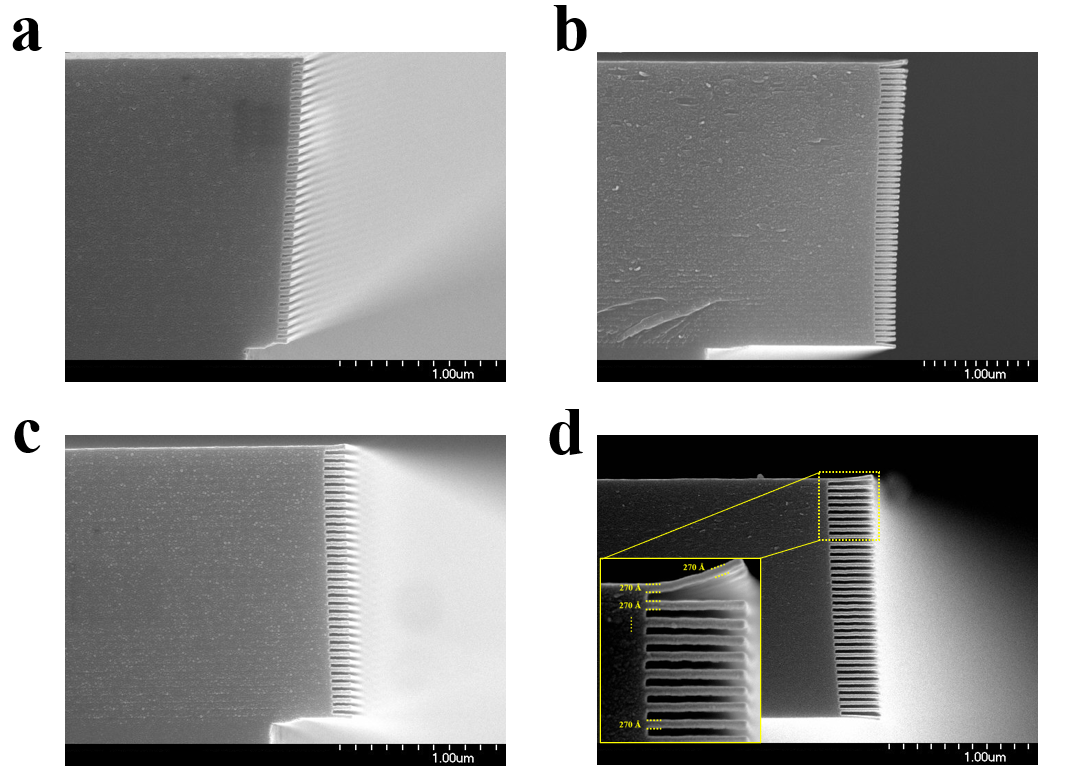


**Figure S3.** Cross-sectional SEM images of SiN_x_/SiO_y_ stacks during the etching with ClF_3_/H_2_ (20 %) plasma for a) 1, b) 2, c) 3, and d) 10 min, respectively.

During the etching of SiN_x_/SiO_y_ stack, the thickness of SiO_y_ was remaining unchanged and no significant change of SiO_y_ thickness between top (which was exposed to the ClF_3_/H_2_ remote plasma directly) and underneath layers was observed up to 10 min etching with ClF_3_/H_2_ (20 %) plasma (subset of figure d)

| Sample | Atomic percentage (%) | | | | | |
| --- | --- | --- | --- | --- | --- | --- |
|  | Si | N | O | F | Cl | C |
| Ref. SiN_x_ | 35.3 | 38 | 19.5 | - | - | 7.2 |
| SiN_x_ (ClF_3_ only) | 33.9 | 33.3 | 12.1 | 15.6 |  | 5.1 |
| SiN_x_ [ClF_3_ & H_2_ (20 %)] | 33.7 | 32.7 | 14.3 | 12.5 | - | 6.8 |
| Ref. SiO_y_ | 32.9 | - | 61.9 | - | - | 5.2 |
| SiO_y_ (ClF_3_ only) | 31 | - | 59.5 | 5.9 | - | 3.6 |
| SiO_y_ [ClF_3_ & H_2_ (20 %)] | 30.5 | - | 59.6 | 5.5 | - | 4.4 |

**Table S1.** Atomic composition of SiN_x_ and SiN_y_ for different ClF_3_/H_2_ etching conditions.

As shown in Table S1, the reference SiN_x_ deposited by PECVD, which is prepared for a stack layer of SiN_x_/SiO_y_ of 3D NAND device, showed the N/Si ratio of 1.07, therefore, it was a Si-rich compound compared with the stoichiometric silicon nitride (Si_3_N_4_; 1.33) while the reference SiO_y_ deposited by PECVD showed O/Si ratio of 1.88 which is close to stoichiometric silicon oxide (SiO_2_; 1:2)
